# Genome-wide comparison of PU.1 and Spi-B binding sites in a mouse B lymphoma cell line

**DOI:** 10.1186/s12864-015-1303-0

**Published:** 2015-02-14

**Authors:** Lauren A Solomon, Stephen KH Li, Jan Piskorz, Li S Xu, Rodney P DeKoter

**Affiliations:** Department of Microbiology & Immunology and the Centre for Human Immunology, The University of Western Ontario, London, Canada; Division of Genetics and Development, Children’s Health Research Institute, Lawson Research Institute, London, Canada; Department of Microbiology & Immunology, Schulich School of Medicine & Dentistry, The University of Western Ontario, London, ON N6A 5C1 Canada

**Keywords:** PU.1, Spi-B, ChIP-seq, Motif, B cell, Gene regulation

## Abstract

**Background:**

Spi-B and PU.1 are highly related members of the E26-transformation-specific (ETS) family of transcription factors that have similar, but not identical, roles in B cell development. PU.1 and Spi-B are both expressed in B cells, and have been demonstrated to redundantly activate transcription of genes required for B cell differentiation and function. It was hypothesized that Spi-B and PU.1 occupy a similar set of regions within the genome of a B lymphoma cell line.

**Results:**

To compare binding regions of Spi-B and PU.1, murine WEHI-279 lymphoma cells were infected with retroviral vectors encoding 3XFLAG-tagged PU.1 or Spi-B. Anti-FLAG chromatin immunoprecipitation followed by next generation sequencing (ChIP-seq) was performed. Analysis for high-stringency enriched genomic regions demonstrated that PU.1 occupied 4528 regions and Spi-B occupied 3360 regions. The majority of regions occupied by Spi-B were also occupied by PU.1. Regions bound by Spi-B and PU.1 were frequently located immediately upstream of genes associated with immune response and activation of B cells. Motif-finding revealed that both transcription factors were predominantly located at the ETS core domain (GGAA), however, other unique motifs were identified when examining regions associated with only one of the two factors. Motifs associated with unique PU.1 binding included POU2F2, while unique motifs in the Spi-B regions contained a combined ETS-IRF motif.

**Conclusions:**

Our results suggest that complementary biological functions of PU.1 and Spi-B may be explained by their interaction with a similar set of regions in the genome of B cells. However, sites uniquely occupied by PU.1 or Spi-B provide insight into their unique functions.

**Electronic supplementary material:**

The online version of this article (doi:10.1186/s12864-015-1303-0) contains supplementary material, which is available to authorized users.

## Background

Development and survival of the B cell lineage is regulated by a number of important transcription factors, and dysregulation of the expression of these proteins can lead to impaired B cell function and disease such as leukemia [1]. Two highly related transcription factors involved in development of B cells are the E26-transformation specific (ETS) family members PU.1 (encoded by *Spi1*, also known as *Sfpi1*) and Spi-B (encoded by *Spib*). PU.1 is expressed in hematopoietic stem cells and common lymphoid progenitors, and throughout B cell development [2]. PU.1-null mice die during late gestation and do not generate progenitors for B cells, T cells, monocytes, or granulocytes [3,4]. Development of lymphoid progenitor cells requires PU.1, making it a key regulator of B-cell fate specification [5]. In comparison, Spi-B deficient mice are viable and develop mature B and T cells, although their B cells are functionally deficient and die in response to BCR signalling [6]. Therefore, PU.1 cannot completely compensate for Spi-B, as functionally deficient Spi-B^-/-^ B cells still express PU.1 at wild-type levels [6].

Several lines of evidence suggest that PU.1 and Spi-B have at least some complementary function in the B cell lineage. First, PU.1 and Spi-B have 43% overall amino acid identity and share 67% amino acid identity within their ETS DNA binding domain [7]. The ETS domains of PU.1 and Spi-B can interact with identical purine-rich 5′-GGAA-3′ motifs and can both activate transcription of a number of genes important for B cell differentiation and function through interaction with the same ETS motifs. Genes with demonstrated regulation by both PU.1 and Spi-B include *Fcgr2b* (encoding FcγRIIb) [8] and *Blnk* (encoding B cell linker protein) [9]. At other genomic regions, binding to the ETS domain may be preferred by one factor over another, particularly at combined ETS-IRF elements where Spi-B is the principal partner for recruiting IRF4 to regulatory regions [10]. PU.1 and Spi-B exhibit differential DNA binding at the *c-fes* promoter in some cell lines, which is predicted to be through the function of distinct activation domains outside of the ETS-binding domain, particularly at the N terminus where there is low homology between the two proteins [11,12].

PU.1 and Spi-B appear to have complementary function in the B cell lineage *in vivo*. Heterozygosity for *Spi1* encoding PU.1 on a *Spib*^-/-^ mutant background leads to defects in BCR signalling and reduced frequencies of splenic follicular B cells that are more severe compared to *Spib*^-/-^ mice, demonstrating a degree functional overlap between PU.1 and Spi-B [13,14]. Conditional deletion of the *Spi1* gene after B cell commitment under the control of the B cell-specific CD19-Cre leads to mild defects in B-cell development and function [4,15,16]. However, conditional deletion of *Spi1* under the control of CD19-Cre on a *Spib*^-/-^ (CD19-CreΔPB) background leads to severely impaired B cell development and B cell acute lymphoblastic leukemia, which is partially attributed to loss of *Blnk* [9,17]. Bruton tyrosine kinase (*Btk*) is also directly regulated by Spi-B and PU.1, and induction of PU.1 in cultured CD19-CreΔPB Pro-B cells restores *Btk* expression and induces apoptosis [18]. While decreased levels of PU.1 and Spi-B are associated with defects in lymphoid development and some forms of leukemia, elevated levels of PU.1 and Spi-B have been demonstrated in lymphoma [19,20]. *SPIB* is amplified in Activated B Cell Diffuse Large B Cell Lymphomas (ABC-DLBCL) compared with other B cell lymphoma subtypes, and is translocated in the OCI-Ly3 ABC-DLBCL cell line leading to over-expression of *SPIB* mRNA compared with other lines [20,21]. Spi-B is required for the survival of ABC-DLBCL cell lines, as depletion of Spi-B using lenalidomide or RNA interference leads to decreased survival *in vitro* [21,22]. It is predicted that the requirement for Spi-B and PU.1 in lymphoma cells is due to an “addiction” to B cell receptor signaling, which is enforced by over-expression of these factors in activated lymphoma subtypes [22].

Next-generation sequencing (NGS) technologies allow for high-resolution analysis and detection of transcription factors across the entire genome. By combining chromatin-immunoprecipitation with high-throughput sequencing, all regions within the genome bound by PU.1 and Spi-B can be identified. Based on the demonstrated complementary function of PU.1 and Spi-B, we hypothesize that PU.1 and Spi-B can interact with the same set of binding sites in the genome of B cells. In this study, we report a genome-wide comparison of genomic regions of interaction by PU.1 and Spi-B in the murine lymphoma cell line WEHI-279. To remove bias introduced by different antibodies, expression levels, or gene regulation we expressed 3XFLAG-tagged PU.1 and Spi-B at similar levels in WEHI-279 lymphoma cells. Chromatin immunoprecipitation was performed using anti-FLAG antibodies. Our results support the hypothesis that PU.1 and Spi-B occupy similar regions within the genome, but also identified a unique subset of regions only occupied by one factor. Additionally, motif analysis has suggested that these regions contain binding regions for different co-activator partner proteins. In summary, these experiments provide biochemical insight into both the similarities and differences between the biological functions of PU.1 and Spi-B.

## Results

### Determination of target regions for Spi-B and PU.1

**Figure 1:**
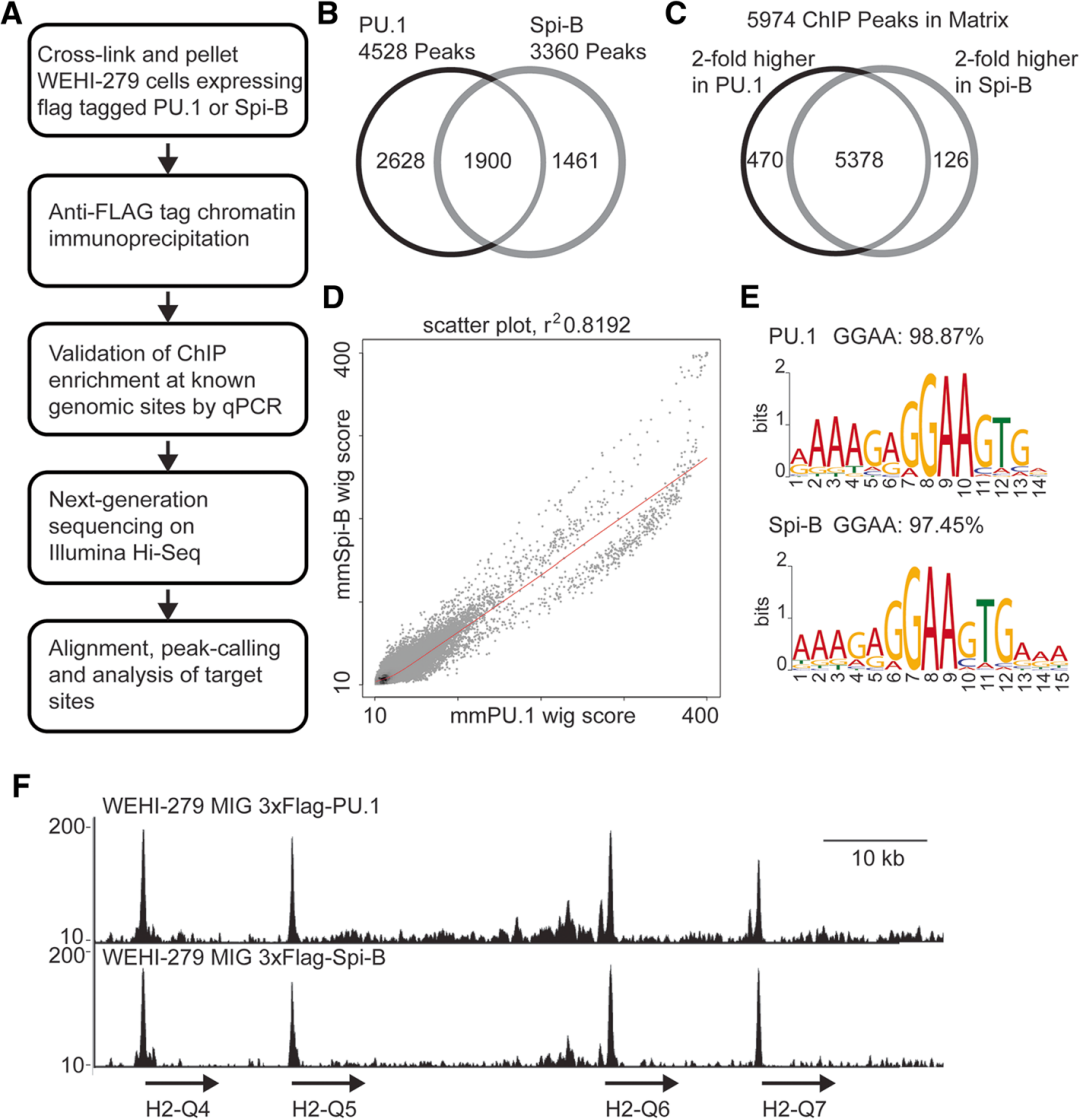
**ChIP sequencing of PU.1 and Spi-B in a mouse B lymphoma cell line. A**. Workflow for generating ChIP sequencing data. **B**. Venn diagram for regions of significant ChIP binding determined by MM-ChIP. Regions with at least 100 base pair overlap were considered common to both factors. **C**. Venn diagram of peakset regions analyzed in DiffBind. The majority of regions in the matrix demonstrated less than 2 fold difference in enrichment between PU.1 and Spi-B. **D**. Scatter plot of ChIP signal between PU.1 and Spi-B binding. Binding of these transcription factors was similar across most of the genome. **E**. De-novo motif analysis from ChIP. From extracted DNA sequences, frequently occurring motifs were discovered using MEME-ChIP. The most frequently occurring motif for each sample contained the canonical ETS binding sequence **F**. Representative ChIP-seq binding at a region considered to be regulated by PU.1 and Spi-B. The histocompatibility 2 Q region on chromosome 17 demonstrates high peaks for PU.1 and Spi-B at gene promoters.

To determine if the transcription factors PU.1 and Spi-B occupied identical regions within the mouse genome ChIP-seq was performed. To ensure a fair comparison, WEHI-279 B lymphoma clones expressing 3XFLAG-tagged full-length PU.1 or Spi-B protein were selected to ensure equivalent levels of protein expression [9]. Uninfected WEHI-279 cells expressed *Spi1* and *Spib* mRNAs at a ratio of 1:1.3, relative to the normalizer gene *Gapdh*. 3XFLAG-PU.1-infected WEHI-279 cells expressed *Spi1* mRNA and *Spib* mRNA at ratio of 1.2:1. 3XFLAG-Spi-B-infected WEHI-279 cells expressed *Spi1* mRNA and *Spib* mRNA at a ratio of 1:2.6. These results suggested that *Spib* mRNA was modestly overexpressed relative to *Spi1* mRNA*.* However, protein levels of the FLAG-tagged products were similar between 3XFLAG-tagged PU.1 and 3XFLAG-tagged Spi-B, as described in Xu et al. [9]. This system is expected to provide the advantage of eliminating variability between antibody specificity and ChIP-Seq yield, while allowing sustained expression of PU.1 and Spi-B in a mouse B cell line. ChIP was performed on fixed WEHI-279 cell lines using anti-FLAG mAb and immunoprecipitated chromatin was validated by qPCR (Figure 1A).

PU.1 or Spi-B binding peaks were determined using replicate data from two experiments, sequenced by two independent sequencing core facilities with over 20 million reads per sample. Replicate ChIP-seq experiments were merged in MM-ChIP, which estimates sample-specific shift-size of ChIP-seq tags with MACS modeling and then pools shifted tags from different samples to identify ChIP-peaks with a dynamic Poisson model [23]. This method allowed combining of separate datasets with different tag-sizes, noise features, and dynamic ranges. Using a model fold of 10, 30 and a p-value cut-off for peak detection of 1e-05, MM-ChIP identified 4528 high-stringency regions bound by PU.1 and 3360 high-stringency regions bound by Spi-B. The PU.1-bound regions called by MM-ChIP had an average size of 1469 bp, while the Spi-B-bound regions called by MM-ChIP had an average size of 1460 bp.

PU.1 and Spi-B-bound regions were compared to determine if significantly enriched regions were common to both sets. Of these 4528 regions, 1900 had a minimum of 100 bp of overlap between the two sets of regions, and the average length of overlap between significantly enriched regions was 1337 out of 1460 base pairs (92%) (Figure 1B). There were also a number of MACS-defined regions that had a PU.1 or Spi-B peak in one sample, but not the other, but did not have a significant 2-fold difference in terms of number of aligned reads. Using DiffBind, 5974 PU.1/Spi-B matrix peak regions were called. 596 high-confidence peak regions were found to differ significantly in tag count between the two conditions (2-fold change, p <0.05). Differentially bound regions were classified as PU.1-higher or Spi-B higher, in which local enrichment of sequence tags for one factor was 2-fold higher than tags for the other. Of these 596 regions, 470 were considered to be PU.1-higher and 126 Spi-B-higher (Figure 1C). The correlation coefficient on the genome scale was calculated using Cis-regulatory Element Annotation System (CEAS) application. Protein-DNA binding signals (Wiggle tracks) were correlated between two samples in 1 kb bins. PU.1 and Spi-B signals demonstrated a high degree of correlation with an r^2^ correlation coefficient of 0.8196 (Figure 1D). These results demonstrated that 1 kb intervals of the genome with a high degree of PU.1 binding were likely to have strong Spi-B binding, and many regions containing significant ChIP-seq peaks for one factor also had a peak for the other. Taken together, these results showed that most regions in the genome of WEHI-279 cells occupied by PU.1 were also occupied by Spi-B.

### Identification of enriched sequence motifs

Members of the ETS superfamily recognize similar purine-rich sequences with a 5′-GGAA-3′ core [24]. To validate the enrichment of ChIP chromatin at ETS motifs, sequences of ChIP-seq peak regions were screened for enriched motifs using MEME, DREME and CentriMO using MEME-ChIP [25]. The top motif for PU.1-bound regions was the Spi1 motif, with an E-value of 8.6e-735, and a central canonical ETS GGAA motif that was found in 98.87% of all peaks. The top motif for the 3360 Spi-B binding sites was also the Spi1 motif with an E-value of 4.1e-398. This motif was centrally enriched contained a central GGAA sequence was found in 97.45% of the peaks (Figure 1E). Top motifs demonstrated central distribution within the sequences.

### Annotation and comparison of peak-associated genes

Potential target genes of PU.1 and Spi-B were identified using BETA-minus, which annotates binding data to predict target genes based on proximity to the transcription start site [26]. To identify and visualize the function of genes potentially regulated by these transcription factors, the predicted target genes from BETA-minus were functionally annotated using **D**atabase for **A**nnotation, **V**isualization and **I**ntegrated **D**iscovery (**DAVID**). Gene ontology analysis of predicted Spi-B targets revealed significant (FDR ≤ 0.05) enrichment of antigen processing functions (GO:0002478, GO:0048002, GO:0019884, GO:0019886, GO:0002495) immune response (GO:0006955), intracellular signaling cascade (GO:0035556) and regulation of lymphocyte activation (GO:0051249). Histocompatibility 2 class II loci, several Fc receptors (*Fcgr2b*, *Fcgr3*, *Fcamr*), as well as B cell markers CD40 and CD74 were included in multiple ontologies.

These gene lists suggest that Spi-B has functions in lymphoid development, antigen processing, and B cell receptor-mediated signalling. Top processes for PU.1 were highly similar to Spi-B, consistent with previous studies that presumptive targets for PU.1 are involved with B cell and myeloid lineages. The gene ontology of processes for PU.1 target genes included cell activation (GO:0001775), leukocyte activation (GO:0045321), and hematopoiesis (GO:0030097). Top genes involved in all three pathways included *Bak1*, *Bcl6*, *Hdac7*, *Pou2f2*, *Spi1*, and *Vav1*. The region upstream of *Spi1* demonstrated a single peak at the -14 kb position (not shown). The region at -14 kb of *Spi1* has been demonstrated to be occupied by PU.1 in both B lymphocytes and myeloid cells, as opposed to a site at -12 kb that is occupied by PU.1 only in myeloid cells [27]. These gene lists are consistent with the known role for PU.1 in both development and function of these lineages [28]. Peak to gene associations for PU.1 and Spi-B are available in Additional file 1: Table S1 and Additional file 2: Table S2, respectively.

To predict genes regulated by either transcription factor, the 1900 regions with more than 100 bp of overlap between PU.1 and Spi-B bound-regions were examined using BETA-minus (Additional file 3: Table S3). Gene targets included the Toll-like receptors *Tlr4*, *Tlr6*, *Tlr9*, and *Tlr13* in addition to well-documented targets such as *Btk* and *Blnk.* Top processes were immune response (GO:0006955), multiple terms for antigen processing and presentation (GO:0002478, GO:0048002, GO:0019884, GO:0019886) and regulation of lymphocyte activation (GO:0051249). These pathways were considered most significant due to high ranking of MHC class II genes in the associated gene list, including H2-Aa, H2-Q4, H2-Q7, H2-Q9 and H2-Q6 (Figure 1F).

### Spi-B and PU.1 ChIP signal at genes highest at transcriptional start sites

**Figure 2:**
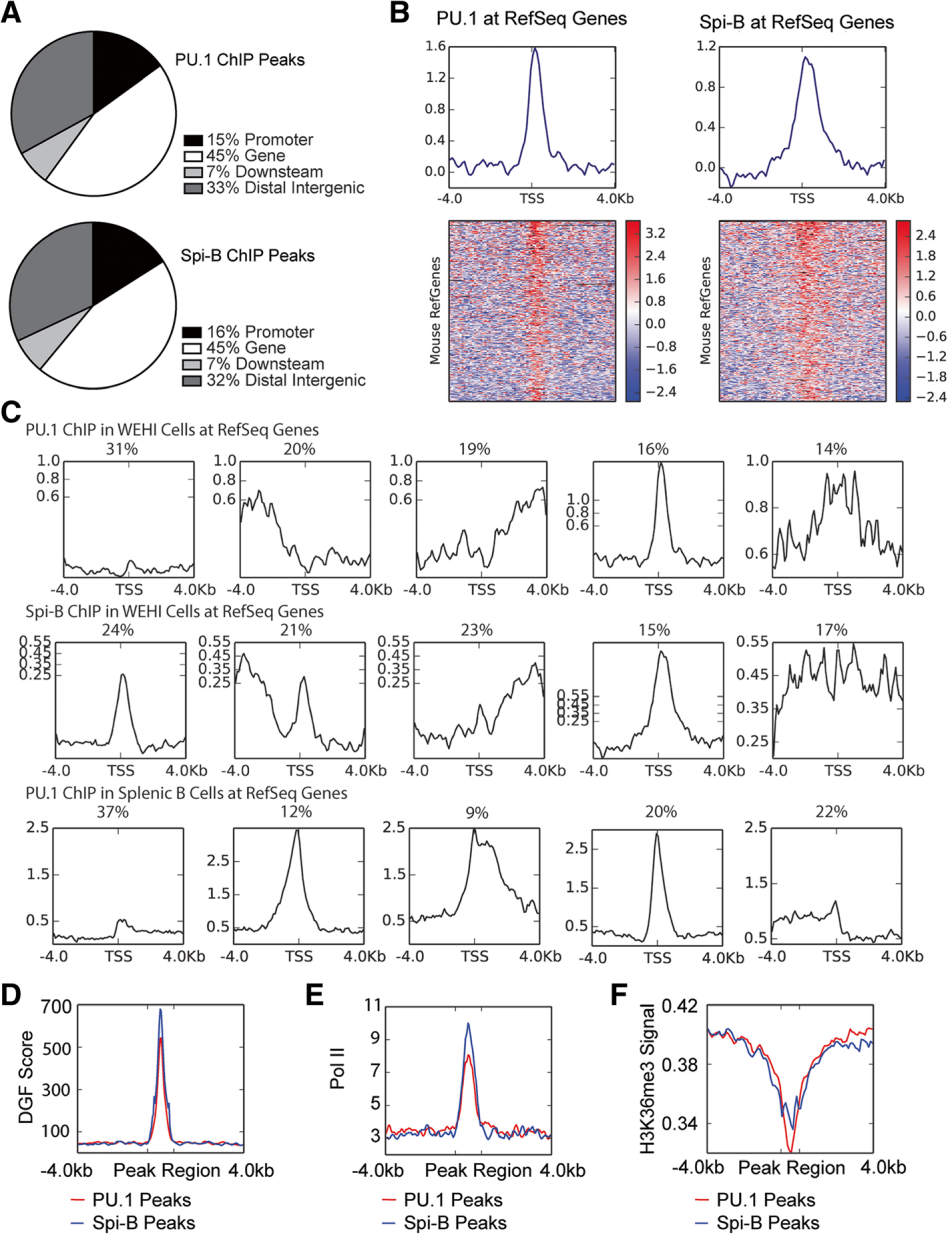
**Distribution of ChIP-seq peaks within the genome. A**. PU.1 and Spi-B have similar binding patterns in relation to features in the genome. **B**. Profiles and associated heatmaps of ChIP-signal at the transcriptional start site of Mouse RefSeq genes. Profiles represent the regions of strongest binding, which are clustered around the transcriptional start site. **C**. All clusters of PU.1 and Spi-B signal in relation to mouse RefSeq genes. Five different patterns of binding were observed, where the largest cluster contained genes without transcription factor binding at the promoter. Clusters 2 and 3 contained genes with binding upstream or downstream of the promoter, although Spi-B binding was often seen at the transcription start site. Cluster 4 contained regions with the highest log2 signal. Cluster 5 was gene with a broad region of transcription factor binding at the TSS. Top row shows results from anti-FLAG ChIP-seq of 3XFLAG-PU.1 in WEHI-279 cells, second row shows results from anti-FLAG ChIP-seq of Spi-B in WEHI-279 cells, and third row shows results from anti-PU.1 ChIP-seq in mouse splenic B cells (GSE21512). **D**. Profile of DNase genomic footprinting (DGF) at regions of significant ChIP enrichment for PU.1 and Spi-B. Regions of PU.1 or Spi-B binding demonstrated increased DNase sensitivity compared to regions outside the peaks. **E**. Profile of RNA Polymerase II ChIP-signal in B cells at regions of ChIP enrichment for PU.1 and Spi-B. PolII data confirmed that significant PU.1 and Spi-B peaks frequently occur at sites of transcription initiation. **F**. Profile of the chromatin feature H3K36me3, a marker of heterochromatin and gene bodies, at peak regions for PU.1 and Spi-B.

In order to visualize genome-wide distribution of ChIP peaks, peaks were sorted according to their distribution around mouse RefSeq genes. *Cis*-regulatory element annotation revealed that regions bound by Spi-B and PU.1 were over-represented for transcription start sites with respect to the whole genome. While 6.6% of the mouse genome is located within 10000 bp of gene promoters, 16.5% of Spi-B bound regions were located within 10000 bp of gene promoters. Similarly, 15.5% of PU.1 bound regions were located within 10000 bp of gene promoters (Figure 2A).

Next, binding profiles of PU.1 and Spi-B relative to features in the mouse genome were determined using DeepTools [29]. Log_2_ normalized read coverage of ChIP-seq data was clustered against regions of mouse RefSeq genes. Regions with the highest PU.1 and Spi-B binding clustered within 4 kb of the transcription start site of mouse genes, with the peak summit directed at the transcription start site and little binding within the gene body (Figure 2B).

Using K-means clustering, transcription factor binding at the start of mouse RefSeq genes in the genome demonstrated 5 distinct patterns (Figure 2C). Cluster 1 contained the largest number of mouse genes, where little PU.1 or Spi-B binding was observed. Cluster 2 demonstrated an upstream binding modality, while cluster 3 demonstrated binding downstream from the transcription start site (TSS). Cluster 4 represented the peaks at the TSS and had the highest signal. Cluster 5 represented broad binding across the TSS, with lower signal intensity than in cluster 4. Importantly, reanalysis of PU.1 ChIP-seq performed using primary splenic B-cells (GSE21512) [30], revealed a very similar enrichment at distal elements and promoters. These clusters suggest that while PU.1 and Spi-B bind various locations within the genome, they have the highest degree of binding at distal elements and promoters of target genes.

In order to further characterize the chromatin environment of regions bound by PU.1 or Spi-B, significant peak regions were profiled against marks for transcription, gene body, and DNase sensitivity. ChIP binding signal in B cells in the form of wig/bigwig files were downloaded from published GEO datasets. Digital genomic footprinting (DGF) of DNase I hypersensitivity in mouse B cells from ENCODE/University of Washington (GSM1003813) was used to predict regions of the genome containing *cis*-regulatory elements profiled against significantly ChIP-enriched regions in the genome (Figure 2D). Regions of the genome bound by either Spi-B or PU.1 demonstrated higher digital genomic footprinting scores relative to regions 4 kb up or downstream. In addition to using DNase footprints for predicted regions of transcription factor binding, promoter regions were further predicted using RNA polymerase binding. Signal for published RNA polymerase II ChIP-seq on stimulated B lymphocytes (GSM1038232) was profiled against ChIP-enriched regions for PU.1 and Spi-B. Similar to DGF profiling, regions of the genome bound by PU.1 or Spi-B demonstrated high enrichment of Pol II binding compared to regions 4 kb up or downstream (Figure 2E).

To further characterize binding locations of Spi-B and PU.1 in relation to the gene body, peak intervals were clustered against histone modifications by ChIP-seq from ENCODE/LICR (GEO series GSE31039). Whole genome coverage of H3K36me3 (GSM1000148) was inversely associated with regions of the genome bound by either PU.1 or Spi-B (Figure 2F). This mark has been observed to be strongest within the body of actively transcribed genes [31]. PU.1 or Spi-B peak regions showed an inverse signal of binding, indicating that regions of PU.1 and Spi-B binding are unlikely to be within regions of the genome also bound by H3K36me3. Taken together, these profiles demonstrate that peak regions of PU.1 and Spi-B are characteristic of transcriptional activators.

### Unique binding sites of Spi-B and PU.1

**Figure 3:**
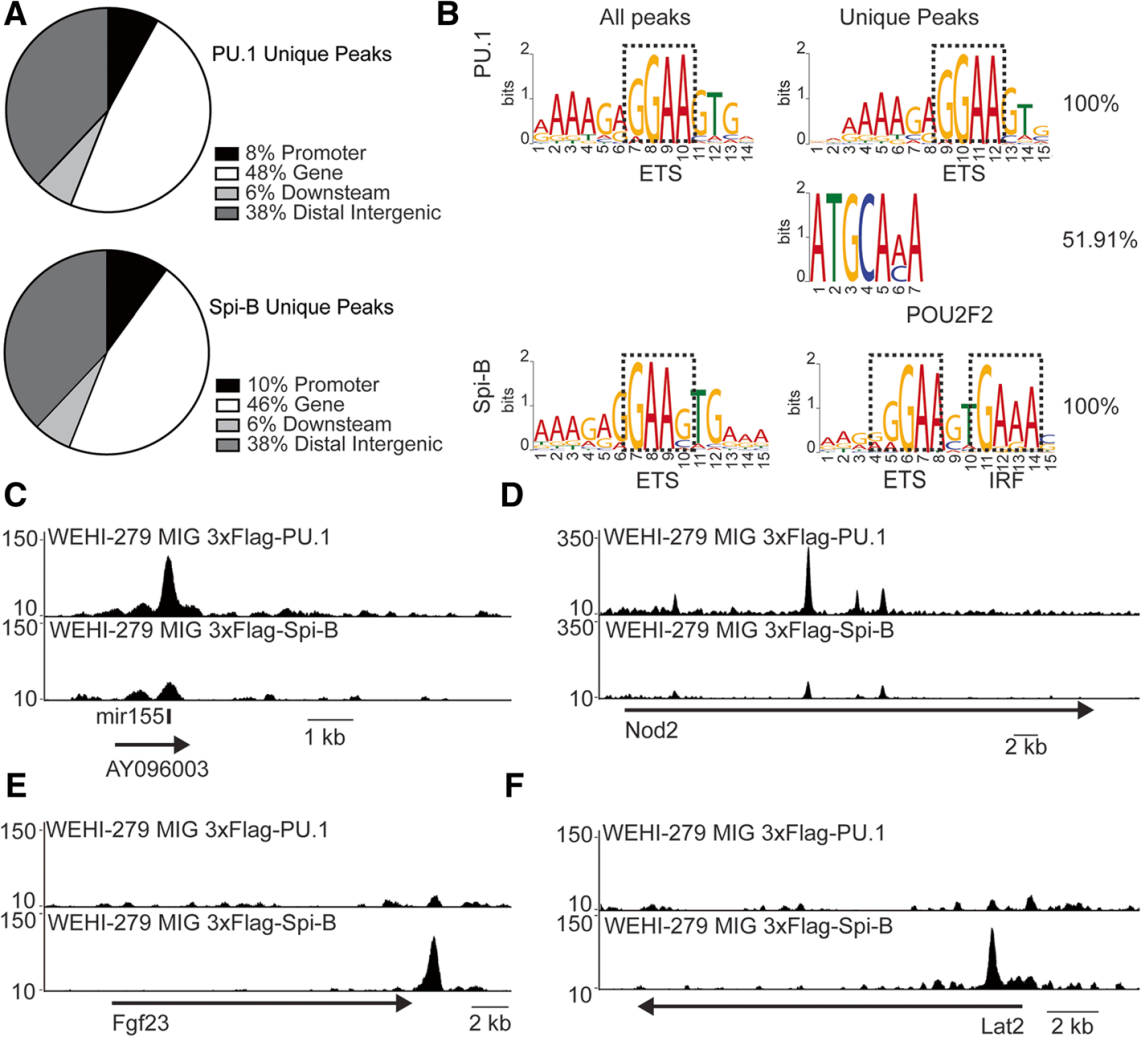
**Differential binding of PU.1 and Spi-B within the genome. A**. Distribution of PU.1 and Spi-B unique ChIP-seq peaks within the genome. Unique sites for these factors are frequently located beyond 10Kb of the transcription start site compared with the overall peak sets. **B**. De-novo motif analysis by MEME-ChIP of unique regions determined by DiffBind. The most frequently occurring motif for each sample is shown and compared with the most common motif found in all peaks for PU.1 and Spi-B. PU.1-unique peaks retained similar motifs to those seen in all PU.1 peaks, but just over half of these peaks also contained a POU2F2 motif. Spi-B unique peaks retained the ETS-IRF motif seen in all Spi-B peaks, with higher conservation of the IRF motif (dotted box). **C**. A unique PU.1 binding peak was observed at the site of mir155 **D**. Unique binding of PU.1 within the gene *Nod2.* **E**. Unique Spi-B binding in the 3′ end of *Fgf23*. **F**. Unique Spi-B binding in the promoter of *Lat2*.

PU.1 and Spi-B demonstrated a consistent pattern of high ChIP signal at transcriptional start sites. Interestingly, binding sites unique to either PU.1 or Spi-B demonstrated distinct profiles. Unlike common peaks, which tended to be associated with TSSs, unique PU.1 or Spi-B sites were frequently found in intergenic regions 10Kb beyond the nearest gene promoter (Figure 3A). Analysis of distribution within the genome using CEAS demonstrated that unique PU.1 peaks were not over-represented at the promoter compared to the background distribution within the genome.

Differentially bound regions were analyzed with MEME-ChIP for unique conserved binding motifs to identify potential differential associated binding partners. Regions uniquely bound by PU.1 contained the ETS motif. A POU2F2 (also known as Oct-2) motif was identified by MEME chip, with an E-value of 2.5e-007. This motif was observed within 100 bp of 19.7% of the ETS motifs seen in PU.1-bound regions, but was overrepresented to 51.9% of ETS motifs in unique PU.1 peaks. There were 267 POU2F2 motif occurrences in unique PU.1 peaks with a p-value less than 0.0001. Combined POU2F2-PU.1 motifs were observed upon re-analysis of PU.1 ChIP in primary splenic B-cells (GSE21512) [30], where a secondary POU2F2 was located in 21.1% of all sequences also containing a GAGGAA primary motif. In contrast, regions bound by Spi-B did not demonstrate a marked difference in POU2F2 motifs. POU2F2 motifs were observed in 12.7% of all Spi-B bound regions and 11.1% of regions uniquely bound by Spi-B but not PU.1.

Remarkably, de-novo motif finding with Spi-B unique regions revealed that 100% of unique Spi-B regions contained an ETS–IRF composite element (EICE; 5′-GGAAnnGAAA-3′) with an E-value of 1.7e-119 (Figure 3B). This combined motif was similar to the binding sequence for all significant Spi-B peaks, but the IRF element was more conserved. The primary GGAAnnGAAA motif was found in 62.38% of Spi-B bound sites. Sites uniquely bound by PU.1 did not demonstrate the same increase in EICEs; GGAAnnGAAA motifs were observed in 52.89% of all PU.1 peaks and reduced to 40.20% in unique PU.1-bound regions. This composite element was observed in Spi-B unique peaks and provides evidence that IRF factors might bind within a few base pairs of the Spi-B binding sequence. To determine whether the uniquely-bound Spi-B sites containing IRF motifs might interact with IRF4, we compared our sites with IRF4 ChIP-Seq regions from mouse B cells (GSE39756) [32]. Of these sites, 30.2% were located within 100 bp of an IRF4-bound site in unstimulated B cells or B cells stimulated with IL-21. 17.5% of ETS-IRF sites were located within 100 bp of an IRF4 peak under both conditions. These findings suggest that unique Spi-B binding is associated with local deposition of IRF4 in B cells.

In order to determine what genes were regulated at differentially bound regions, gene function for differential gene targets was determined using gene ontology. Peak to gene associations were determined using CEAS and annotated using DAVID. Genes uniquely bound by PU.1 were predominantly associated with leukocyte activation during immune response (GO:0002366) and cell activation during immune response (GO:0002263) (Additional file 4: Table S4). These pathways were both highly ranked due to inclusion of interleukin 3 and 12b, *Cx3cr1*, as well as a unique peak in an intron of *Nfkb1* not observed in Spi-B. PU.1 unique peaks were found to be associated with genes related to mRNA processing (GO:0006397) and with a number of miRNAs; mir-155/AY096003, mir-3108/zfhx3, mir-1938/Ifrd1, mir-3102/Arhgef17, and mir-5130/Kctd12. Of these, mir-155 has a known oncogenic role in lymphomas [33]. Furthermore, mir-155 directly targets PU.1 and leads to a down-regulation of PU.1 protein in B cells [34]. PU.1 unique peaks were observed in the promoter region of *Nod2*, a NOD-like receptor involved in B cell proliferation and activation [35]. Typical examples of these regions are represented in Figure 3C,D.

Gene targets of differentially bound Spi-B peaks were associated with immune effector process (GO:0002252) and B cell activation (GO:0042113) that included *Bnip3l*, *Lat2* and *Pou2f2*. Multiple GO terms associated with both positive and negative regulation of transcription and gene expression (GO:0045935, GO:0045499, GO:0032583, GO:0008634) were also identified due to the inclusion of *Fgf23*, *Myc* and *Pou2f2* (Additional file 5: Table S5). A large unique peak of Spi-B binding was observed at a combined ETS-IRF motif within the 3′ end of *Fgf23* (Figure 3E). FGF23 is involved in the regulation of phosphate concentration in plasma, and has been found to be elevated in the serum of patients with B cell neoplasms [36]. Spi-B unique binding was also observed at the promoter of *Sema4b*. Sema4b abundantly expressed in lymphocytes and been demonstrated to be a novel regulator of immunological memory responses and homeostatic T helper 1/T helper 2 balance in mice [37]. *Lat2* (also known as Lab/NTAL) (Linker For Activation Of T Cells 2) demonstrated significant Spi-B binding directly at the promoter, which was not observed in the PU.1 ChIP (Figure 3F). *Lat2* is expressed in the spleen, peripheral blood lymphocytes, and various human B cell lines, and is tyrosine-phosphorylated in response to B cell receptor signalling [38]. Lat2 expression has been observed in monocytes (CD14^+^) and B cells (CD19^+^) [39]. These analyses demonstrate that while PU.1 and Spi-B frequently occur at the regulatory regions of genes predicted to have B cell function, a small subset of regions and genes that are uniquely bound by only of these transcription factors. These factors have unique requirements for associated binding partners, and are involved in different functions.

## Discussion

To investigate genome-wide binding of Spi-B and PU.1, we conducted next generation sequencing of chromatin immunoprecipitated with anti-3XFLAG-tagged Spi-B or PU.1. Previous studies have reported ChIP-seq analysis of PU.1 binding sites in erythroid, myeloid, and lymphoid cells but none have compared PU.1 to Spi-B sites in mouse cells due to technical constraints [30,40,41]. Detailed analysis of transcription factor binding revealed that these two ETS transcription factors had similar binding regions within the genome of murine WEHI-279 lymphoma cells. We determined that both transcription factors demonstrated the highest degree of binding near the TSS and regions of open chromatin, although peaks were also observed within introns and upstream of genes beyond 10 kb. Characterization of the sequences most strongly bound by PU.1 and Spi-B has confirmed that the primary target sequence of both factors contained the purine-rich core motif 5′-GAGGAA-3′. This motif was identified within regions of significant ChIP enrichment, and determined to be overrepresented by de-novo motif enrichment analysis. Peak-to-gene associations confirmed occupancy of both factors within 15,000 bp of the TSS for genes involved in immune system regulation and processes. Gene ontology analysis confirmed that PU.1 and Spi-B are involved in the regulation of genes required for B cell development and function. The top GO pathways determined by DAVID for PU.1, Spi-B, and the common peaks were cell activation, immune response and immune response, respectively.

An important finding of this study is that of the genomic regions occupied by PU.1, a majority were also occupied by Spi-B. This fraction was 1900/4528 regions using MM-ChIP, and 5378/5974 regions (90%) using DiffBind. This result supports our hypothesis that PU.1 and Spi-B can interact with the same regulatory regions in the genome of B cells. This result is consistent with previous reports that PU.1 and Spi-B have similar DNA binding specificities [12,24]. We suggest that similar DNA binding preferences and similar biochemical activity are the explanation for why PU.1 and Spi-B have complementary biological activity in the B cell lineage [14,17,42].

Fewer regions of the genome were found to be differentially occupied by PU.1 or Spi-B in the genome of WEHI-279 B cells. One important aspect of next-generation sequencing demonstrated in this study is the importance of mathematical modeling and input sequencing in identifying differential peaks. While direct subtraction of regions called by peak callers revealed several peak regions that did not overlap between samples, affinity (quantitative) data analysis using DiffBind determined that only several hundred regions demonstrated significant differences between factors. Using DiffBind to identify differential peaks in combined data most likely under-predicted gene targets, but provided high-stringency lists. Therefore, while there remains a possibility that other differential regions exist within the genome, this method provides the most accurate and specific regions. Therefore, subsequent analysis of these high-stringency peaks highlights the most significant differences between Spi-B and PU.1 binding within the genome.

Regions identified that were occupied uniquely by PU.1 or Spi-B may reveal insights into biological functions unique to each factor. Genes associated with unique PU.1 bound regions were related to immune development using Gene Ontology analysis. Regions unique to PU.1 contained a number of microRNA genes, while no microRNA genes were observed at unique Spi-B peaks. The unique binding of PU.1 to the sequence of MiR-155 may have a role in regulation of PU.1 itself, as expression this micro-RNA has been shown by others to down-regulate PU.1 [34]. Binding at this region suggests that PU.1 may be involved in the regulation of MiR-155. The identification of an Oct2 binding motif within unique PU.1 regions implies a potential co-activator role, as PU.1 and Oct2 have previously been shown to bind concomitantly at the Vκ19 promoter in B lymphocytes, and point mutations in the binding sites for either factor can lead to diminished immunoglobulin production [43]. Oct2 binding has been shown to be covariant with PU.1 binding activity [44] and Oct2 preferentially binds to PU.1 regions in B cells compared with macrophages, suggesting a functional role for PU.1 to recruit Oct2 to regulatory regions of the genome and induce cellular reprogramming in B cells [30].

Unique Spi-B associated genes were associated with roles in B-cell activation and immune function. All unique Spi-B peaks had an associated IRF sites in WEHI-279 lymphoma cells, while ETS-IRF sites were not overrepresented in unique PU.1 peaks. This suggests that in cases where genes are under the regulation of an ETS-IRF composite element (EICE), Spi-B and not PU.1 is the preferential IRF partner. Our results are consistent with a recent study showing that combined EICEs were dominantly occupied by Spi-B in B lymphoma cell lines, and that PU.1 could not fully compensate for Spi-B in recruiting IRF4 to target regions [10]. RNAi-mediated down-regulation of either Spi-B or IRF4 leads to rapid death of cultured lymphoma cells, irrespective of PU.1 co-occupancy [22]. Therefore, Spi-B may be involved in a unique regulatory network where it is more frequently associated with IRF4 in B cell lymphomas, and may be directed to these sites by IRF4 or function to alter IRF4 occupancy within the genome.

Our model of PU.1 and Spi-B binding is based on lymphoma cells over-expressing the proteins, which bears similarity to other B cell studies in terms of binding near target genes and associated binding motifs. While the changes in the expression level of PU.1 can enhance binding of other factors to influence local chromatin structure and dictate eventual cell fate [27,30,45,46], our tagged-expression model does not appear to have substantially altered binding of PU.1 or Spi-B, since identified peaks were very similar to those identified in a study of mouse splenic B cells [30]. Peak-associated gene lists support our hypothesis that PU.1 and Spi-B are involved in the regulation of lymphoid development and immune function, and we report fewer than 10,000 peaks per factor, possibly due to the restricted lineage of the lymphoma cell line.

## Conclusions

In conclusion, we report genome-wide binding of the ETS transcription factors Spi-B and PU.1 to similar regions of the genome, with high affinity for transcriptional start sites containing ETS motifs. These regions are frequently located near the regulatory domains of genes involved in B cell development and function. In addition to these common regions, there exists a small set of unique regions where PU.1 and Spi-B demonstrate differential preference for associated factors based on sequence identity, target gene function, and potential co-activators. Our findings provide novel insight into differences between the two factors, and offers novel biochemical pathways involving Spi-B as a molecular targeting therapy in B cell malignancies.

## Methods

### Quantitative PCR (RT-qPCR)

Relative frequencies of Spi-B and PU.1 mRNA transcripts were measured by reverse transcription–quantitative PCR (RT-qPCR) and normalized as a percent of *Gapdh* transcripts using the comparative threshold cycle method [47].

### Chromatin immunoprecipitation

Mouse WEHI-279 lymphoma cells were cultured in complete DMEM medium (Multicell, 4.5 g/L glucose) supplemented with 10% fetal bovine serum, 1x penicillin/streptomycin/L-glutamine, 5 × 10^-5^ β-mercaptoethanol and 5 mM HEPES buffer. Cells were stably transfected to express 3x FLAG-tagged Spi-B or PU.1 as previously described [9]. Chromatin was cross-linked in 1% formaldehyde for 10 minutes and quenched with 0.125 M glycine. Cell pellets were washed 3 times in cold PBS and snap frozen in liquid nitrogen. Chromatin fragmentation was conducted using 30 cycles of sonication on a Bioruptor (Diagenode). PU.1/Spi-B-DNA immunoprecipitation was performed using anti-FLAG microbeads (M2, Sigma-Aldrich) on 300 ug of chromatin per sample. Cross-linking was reversed at 65°C overnight and DNA isolated used a QIAquick PCR purification kit (Qiagen). Fragmented DNA was quantified using 2100 Bioanalyzer (Agilent Technologies). Libraries were generated robotically with 10 ng of fragmented DNA (100-300 bp) using the Kapa HTP Library Preparation Kit (Kapa Biosystems) as per the manufacturer’s recommendations except that adapters and PCR primers were diluted 100-fold, the size selection step was done after the PCR step and the number of PCR cycles increased by 6. Adapters and PCR primers were purchased from Integrated DNA Technologies whereas size selection has been performed on a Pippin Prep instrument (SAGE Biosciences Inc). Libraries were quantified using the Quant-iT™ PicoGreen® dsDNA Assay Kit (Life Technologies) and the Kapa Illumina GA with Revised Primers-SYBR Fast Universal kit (D-Mark). Average size fragment was determined using a LaChip GX (PerkinElmer) instrument. Libraries were sequenced on a SR100 run on a HiSeq2000 (Illumina).

### ChIP-seq analysis

ChIP-seq experiments were conducted in duplicate for each transcription factor. Peak finding and data analysis was performed using Galaxy Suite [48,49]. FASTQ files were aligned to MM9 using Map with Bowtie for Illumina v. 1.1.2 to the reference genome (NCBI37/mm9) [50]. Peaks were called using MACS version 1.0.1 [51] with a mappable genome size of 1910000000 bp (MM9). Input controls were used when available. Peaks were called with a tag size was set to either 75 (rep1) or 100 (rep2), band width of 300 and a P-value cutoff for peak detection of 1e-05. Peak Model was generated using an MFOLD high-confidence enrichment ratio against background of 15. Wiggle files were created using a resolution of 10 bp. Independent experiments were combined using MM-ChIP v. 1.0.0 with a mappable genome size of 1910000000, band width of 200, model fold = 10,30 and a p-value cut-off for peak detection of 1e-05 [23]. Larger datasets were scaled towards smaller datasets. Regional lambda was calculated at a range of 1000 bps and 10000 bps. Input .bed files of total reads for MM-ChIP were generated using Convert from BAM to BED tool v0.1.0 in Galaxy. Functional analysis of cis-regulatory regions bound by PU.1 and Spi-B were identified using CEAS [52]. Promoter regions were defined as regions extending 10 kb upstream of transcription start site. Peak coverage was compared by subtracting intervals of Bed files and reporting intervals that did not have at least 100 bp overlap between datasets using Intersect (version 1.0.0) in Galaxy.

### Peak to gene associations

Peak to gene associations were determined using BETA-minus in Cistrome [52]. BED files for peaks were generated in MM-ChIP, or DiffBind for differential peaks. All peaks were considered to contribute to genes. Parameters set were: Genome = mm9, bl = false, Distance = 15000. Gene ontology was determined using DAVID [53,54]. Gene lists were submitted for functional annotation as OFFICIAL_GENE_SYMBOL and process results reported from the GOTERM_BP_FAT chart.

### Motif analysis

Enriched motifs within the ChIP-seq peaks were determined using MEME-ChIP [25]. Peak regions were converted to FASTA sequences using Extract Genomic DNA (version 2.2.3) in galaxy (mm9). Peak regions were submitted for motif discovery in MEME-ChIP using JASPAR vertebrates and UniPROBE mouse. Site distribution was expected to be any number of repetitions, with a minimum width of 4 and a maximum width of 30.

### Heat mapping and gene profiling

Binding profiles of Spi-B and PU.1 relative to features in the mouse genome were depicted using DeepTools [29]. Normalized read coverage (log_2_ratio) of the ChIP-seq data was clustered against the intervals of all Mouse RefSeq genes (33,073 regions) using computeMatrix v. 1.5.8. RefGenes were plotted using reference-point mode, with the beginning of each gene region designated as the transcription start site. Heatmapper images were generated, with regions sorted in descending order. K means clustering was set to 5, and individual profiles generated from each cluster. To profile GEO datasets against declared peak regions, computeMatrix was run using signal from published datasets and significant ChIP peaks from this study as the regions of interest in scale-regions mode. All peak regions were scaled to 1500 bp for signal profiling. Signal was calculated across the region, plus 4 kb upstream from the peak start site and 4 kb downstream of the peak end site. Scores were averaged over 50 base pair bins. Indicate missing data as zero: False, Skip zeros: False. Signal files of ChIP-seq in B cells were downloaded from GEO: DNaseI Hypersensitivity by Digital DNaseI from ENCODE/University of Washington (GEO Series GSE37074). Whole genome coverage of DNaseSeq in CD43- B cells (GSM1014170). ChIP-seq from ENCODE/LICR (GEO series GSE31039). Whole genome coverage of H3K36me3 in CD43- B cells (GSM1000148). ChIP-seq for serine 5-phosphorylated RNA Polymerase II in Wild-type B cells (GSM1038232). For GEO datasets, all files were downloaded as pre-analyzed wig/bigwig files and used directly for profiling. Profiles were generated using the Matrix file from computeMatrix in profiler, combining both regions plotted on the same plot.

### Availability of supporting data

The data sets supporting the results of this article are available in the GEO repository, http://www.ncbi.nlm.nih.gov/geo/query/acc.cgi?acc=GSE58128. Additional data files supporting the results of this article are included as Additional file 1: Table S1, Additional file 2: Table S2, Additional file 3: Table S3, Additional file 4: Table S4 and Additional file 5: Table S5.

## Additional files

Additional file 1: Table S1. A supplemental table containing peak-to-gene association of PU.1 to mouse RefSeq genes. Significantly enriched ChIP regions bound by PU.1 were associated with genes based on proximity using BETA-minus. Additional file 2: Table S2. A supplemental table containing peak-to-gene association of Spi-B to mouse RefSeq genes. Significantly enriched ChIP regions bound by Spi-B were associated with genes based on proximity using BETA-minus. Additional file 3: Table S3. A supplemental table containing peak-to-gene association of regions with at least 100 bp of overlap between PU.1 and Spi-B to mouse RefSeq genes. Significantly enriched ChIP regions bound by both regions were associated with genes based on proximity using BETA-minus. Additional file 4: Table S4. A supplemental table containing peak-to-gene association of regions uniquely bound by PU.1 to mouse RefSeq genes. Significantly enriched ChIP regions with unique PU.1 binding were associated with genes based on proximity using BETA-minus. Additional file 5: Table S5. A supplemental table containing peak-to-gene association of regions uniquely bound by Spi-B to mouse RefSeq genes. Significantly enriched ChIP regions with unique Spi-B binding were associated with genes based on proximity using BETA-minus.

## Abbreviations

ChIP: Chromatin immunoprecipitation
CEAS: Cis-regulatory element annotation system
DAVID: Database for annotation, visualization, and integrated discovery
DGF: Digital genomic footprinting
ETS: E26-transformation specific
EICE: ETS-IRF composite element
GO: Gene ontology
IRF: Interferon regulatory factor
NGS: Next generation sequencing
TSS: Transcription start site
